# Assessment of water, sanitation, and hygiene among women living in precarious households in a Turkish urban area

**DOI:** 10.1186/s12905-023-02861-8

**Published:** 2024-01-03

**Authors:** Sera Şimşek, Zeliha Aslı Öcek, Meral Türk, Ayşegül Ünver

**Affiliations:** 1 Postdoctoral Researcher, Public Health Department, Koc University Faculty of Medicine, İstanbul, Turkey; 2 Chair of Public Health and Health Services Research, Institute for Medical Information Processing, Biometry and Epidemiology, Pettenkofer School of Public Health, Ludwig-Maximilians-Universitaet, Munich, Germany; 3 Parasitology Department, Ege University Faculty of Medicine, İzmir, Turkey; 4 Public Health Department, Ege University Faculty of Medicine, İzmir, Turkey

**Keywords:** Water, Sanitation, Hygiene, Household, Poverty

## Abstract

**Background:**

This study aimed to identify the determinants of water, sanitation, and hygiene (WASH) behaviors and conditions among women in poor neighborhoods in Izmir, Turkey, and to develop a scale for assessing WASH behaviors and conditions that is specifically designed for use in precarious urban areas.

**Methods:**

The study used a cross-sectional design, as well as a methodological feature for developing the scale. The sample size was calculated as 243 households out of 2667 households in the Basmane neighborhood, with a 95% confidence interval and a 6% margin of error, and a woman who was responsible for cleaning was invited to participate from each household. The scales for WASH behaviors and conditions, which served as dependent variables, were developed in a four-stage process, yielding two distinct scales. The WASH-Behaviors Scale had 14 items about hand, body, and home hygiene, whereas the WASH-Conditions in Households Scale included 16 items about variables like area per capita, physical structure, and cleaning tool availability. Age, ethnicity, number of children, education, work status, and income were among the independent variables. Data was collected through household visits. The scales’ validity was evaluated using exploratory factor analysis. Linear logistic regression analysis was employed to assess the determinants of WASH behaviors.

**Results:**

The women, with an average age of 40.65 ± 14.35 years, faced economic challenges, as a substantial portion earned an income below the minimum wage. More than half of them were uninsured, and 72.6% were identified as migrants or refugees. Factor analysis confirmed the compatibility of both scales (KMO = 0.78–0.80, *p* < 0.05), elucidating 52–54% of the total variance. Factors such as ethnicity, number of children, husband’s education level, income perception, and WASH conditions explained 48% of WASH behaviors.

**Conclusions:**

WASH-Behaviors and WASH-Conditions in Households scales met the validity criterion, and their scores were related to basic sociodemographic and economic characteristics like education, income, household size, and ethnicity. The scale development process emphasized the importance of considering both behaviors and household conditions, albeit using different techniques. The findings indicated that WASH conditions are more problematic than behaviors, and that behavioral interventions will not work unless the conditions are corrected.

## Introduction

Poverty, which can be defined as the state of lacking the essential resources necessary for a standard way of life [1], is further intensified in urban areas characterized by precarious living and working conditions [2]. Discrimination, crowded environments, exposure to air and water pollution, a lack of access to safe water and food, sanitation facilities, and healthcare are all features of urban life for vulnerable groups [3]. The urban poor have higher rates of total fertility, communicable and non-communicable diseases, and maternal and child mortality ratios [4]. The COVID-19 pandemic has highlighted these aspects of urban poverty that inevitably lead to poor health [3].

Every year, at least one million children die from diarrheal diseases due to the vicious circle of poverty and a lack of safe water, sanitation, and hygiene (WASH) facilities [5]. Therefore, continuity of basic WASH services is essential for the prevention and control of infectious diseases such as COVID-19 as well as the fight against urban poverty [4]. Intervention programs that combine improved access to safe water, sanitation infrastructure, and hygiene practices have been effective in lowering poverty-related health issues like diarrheal diseases, stunting, and malaise in young children [6–8]. The program’s achievements have illustrated the necessity of recognizing these three crucial health determinants as a unified structure. The limited success and discontinuous effects of programs focusing solely on behavior change also point to the need for a more comprehensive approach [9]. Furthermore, given the strong link between urban poverty and the triad of water, sanitation, and hygiene, the issue should be approached from the standpoint of structural and social determinants that make people vulnerable. On the other hand, comprehensive tools are required to consider poverty and inequality beyond simple WASH interventions. The present research has been planned to assess these three core issues, namely water, sanitation and hygiene with a holistic approach in Izmir, a Turkish city where deep inequalities and urban poverty are experienced [10].

75% of Turkey’s population lives in cities, with a sizable proportion of the urban population made up of the poor, migrants, and various minorities employed in the informal sector [1]. The Izmir Municipality is planning interventions to address the social determinants that make residents of the district vulnerable. In order to guide these intervention programs, it was decided to assess the needs in neighborhoods with precarious housing and living conditions. Due to the lack of an integrated tool, it was decided to create one capable of evaluating WASH in the context of socioeconomic determinants. The first aim of this research was to identify WASH status in the Basmane region, as well as thesocioeconomic determinants of WASH behaviours and conditions. The second aim was to develop a scale to assess WASH behaviors and conditions that can be used in impoverished urban areas.

## Material and method

### Design

The main aim of the study utilized a cross-sectional design, while the secondary aim, which involved the development of a scale, followed a methodological design. Approval from the Ege University Medical Research Ethics Committee was obtained (E.8515). The study encompassed a duration of 18 months, commencing in April 2021 and concluding in September 2022.

### Study setting and population

. The Basmane district of Izmir city exemplifies urban poverty, exhibiting various infrastructural difficulties, precarious housing conditions, the existence of slums, and a high population density. The region’s proximity to the primary train station of the city has led to its hosting of a population characterized by a historical pattern of migration from Turkey’s most economically disadvantaged regions as well as nations, including Afghanistan, Syria, and Iraq 4,11. According to data available, 67% of the Basmane population is unregistered and works in small-scale manufacturing or in marginal activities such as day labor and illegal street vending. One-third of the region receives social assistance, while one out of every two people is uninsured [10].

Before starting the study, the first author (SŞ) visited the area three times to gather information about the living conditions and gain the trust of the community as a physician by providing counseling on various health issues. Meanwhile, the people she spoke with insisted that women oversaw WASH in the home and that she would be unable to obtain information on the subject from men. Women representatives from five different municipality-community centers, as well as the municipality’s director of health affairs, shared the same viewpoint. As a result, it was decided that only women would be included in the study. There were no exclusion criteria for the women in the study; all women who were both housewives and working were included.

During a meeting, two municipal administrators in charge of health and social affairs, along with a social worker with 15 years of experience in the area identified a region that included socioeconomically disadvantaged neighborhoods that were deemed appropriate for the research objectives. Subsequently, the boundaries of this region, which included 2667 households, were delineated on a sketch. The sample size was calculated as 243 households with a 95% confidence interval and a 6% margin of error. The research utilized a systematic sampling methodology, in which the selection procedure began with the closest residence to the community center, followed by the visitation of every tenth house thereafter. During the course of the visits, the first author (SŞ) conducted a comprehensive evaluation of each household through direct observation, specifically emphasizing the identification of shared spaces such as the kitchen, toilet, and inner courtyard. An interview was conducted with women over the age of 18 who live permanently in the household andwere the primary individual responsible for housekeeping.

In 12% of the households, a person who met the criteria could not be reached, an Arabic translator could not be found, and/or permission for household visits was denied. As a result, 215 households were included in the study, representing 88% of the initial sample size and %86 power.

### Scale development process

We developed a scale for the study’s dependent variable, the WASH level. The four-stage development process began with the creation of a WASH-condition, household hygiene, and personal hygiene behavior item pool [9, 12–14]. WASH conditions include whether the toilet is private or shared, if it is outside or inside the house, if soap and toilet paper are always available, and other general household conditions. Hand washing frequency in various situations, such as before preparing food, laundry washing practices (frequency and temperature), brushing teeth at least once a day, changing underwear and showering frequency, household waste disposal practices, and house cleaning are all examples of WASH behaviors.

**Fig. 1:**
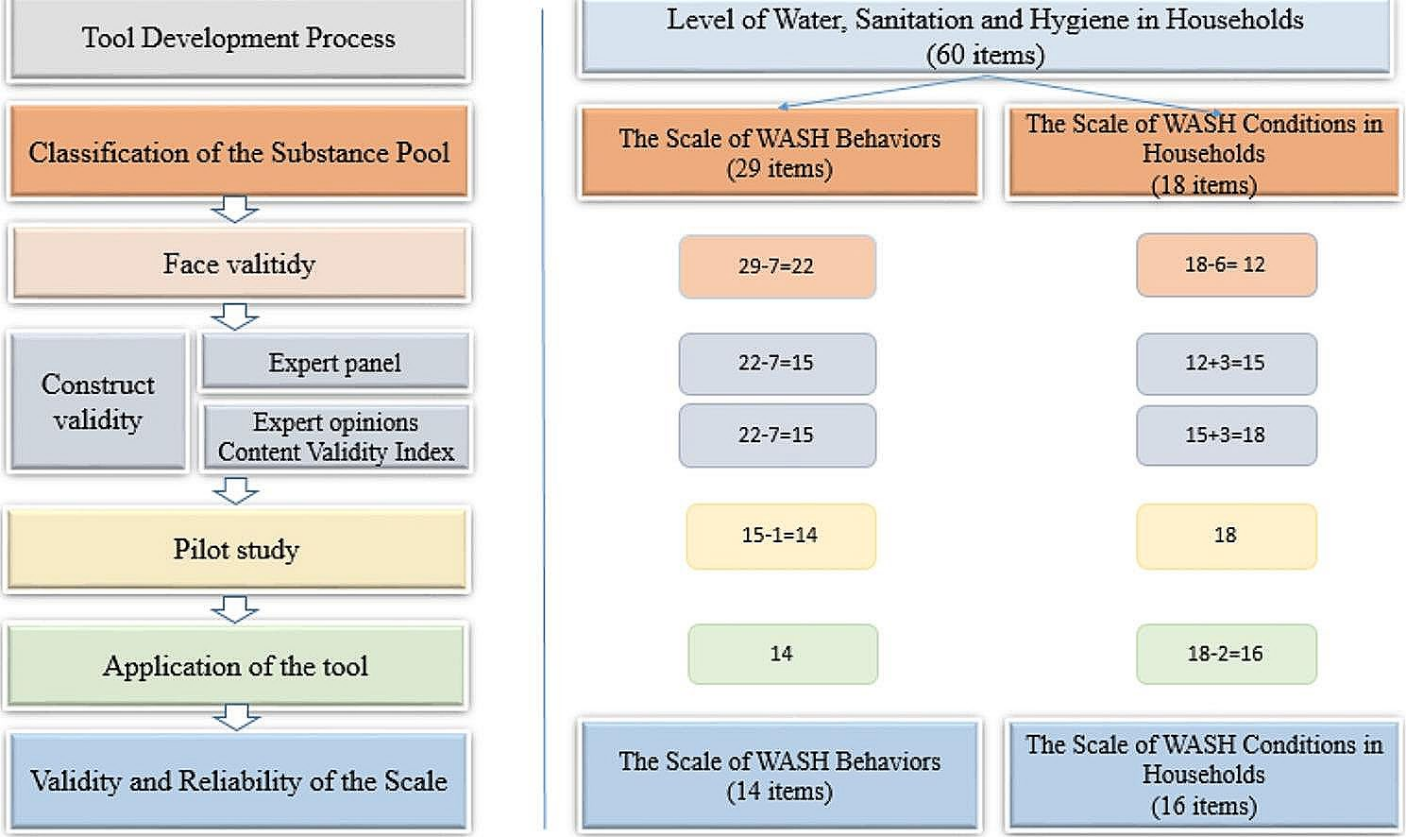
The development process of scales

The items were evaluated for face validity in the second stage at a meeting attended by three public health specialists and a medical parasitologist, and it was decided to develop two separate scales measuring behaviors and conditions rather than a single comprehensive one. Expert opinions were obtained in the third stage to assess the content validity. Each item was evaluated by 16 experts (1-not relevant, 2-item needs to be changed appropriately, 3-quite relevant but minor changes are needed, 4-very relevant). Accordingly, Content Validity Ratio (CVR) values which were used to quantify the content validity of measurement scale for each item were found to be greater than 0.54 and the items were considered to have content validity [15]. The expert panel of 11 people (public health specialists, social workers, parasitologists, psychologists, family physicians, and municipality physicians) was assembled for the fourth stage, at which point it was decided to base the first form’s determination on the statements of the subjects and the second form’s on the investigators’ observations. The fourth stage involved conducting a pilot study on ten households in a neighborhood that was relevant to the study population’s socio-demographics. As a result of necessary additions and deletions to the items at each stage, a 14-item scale of WASH-behaviors and a 16-item scale of WASH-conditions were developed (Fig. 1).

### Independent variables

- Age, ethnicity, mother tongue, nation of birth, marital status, number of children, monthly income level, perceived income of the household, education and employment status of the woman and her husband, spouse’s social security, total number of occupants, diarrhea, and parasite infection in the household were independent variables. The term “mother tongue” refers to the language most often used by family members to communicate in the home where a person was born and raised, and the term “ethnic group” refers to the name of the group that defines the household. Relative household income was defined as how a person determines his/her own income level in relation to society and peers. A doctor’s diagnosis of a household member having a parasitic infection within the previous three months or experiencing watery diarrhea 3–4/day were positive cases.

### Data collection

In addition to the scales, a questionnaire on the independent variables was used. The first author (SŞ) conducted home visits in the company of female community leaders. The interviews were conducted in Turkish or Kurdish, with the assistance of volunteer Arabic translators from the Refugee Center. After conducting face-to-face questionnaires with the woman who was primarily responsible for housecleaning, the first author evaluated the Conditions in Households scale by observing the internal and external structure. Women who were unable to be at home due to work or other reasons were visited again at times when they were at home based on information provided by family members or neighbors. The data was collected in two months (September and October).

### Statistical analysis

The reliability of the WASH-Behaviors scale and the WASH-Conditions in Household scale was determined using Cronbach’s coefficient, while the validity was determined using exploratory factor analysis (oblimin) and the Spearman-Brown split-half, item-to-total correlation method. The suitability of the scale items for factor analysis and their correlations with each other were investigated using KMO and Barlett sphericity analyses. The total score of the WASH-Behaviors scale was found to be compatible with the normal distribution using the central limit theorem, whereas the total score of the WASH-Conditions in Households scale was found to be incompatible. To determine the factors associated with the scores of both scales, Student T test, Mann-Whitney U test, Kruskal Wallis test, and ANOVA test were used. A multiple linear regression analysis with WASH-Conditions as a variable was performed to specify the factors influencing WASH-Behaviors.

## Result

The participants had a mean age of 40.65 ± 14.35. (min:19, max:84). The proportion of women born in Syria was 38.6%, while 34.9% of women reported Arabic as their mother tongue. The vast majority (74.9%) were homemakers (Table 1). The average household income was less than the minimum wage and 54.4% of the households reported having a negative perception of their income, more than half of them were uninsured. At least five people resided in two-thirds 44.7% of the homes. One in ten households had a parasitic disease, and one in four individuals had diarrhea.

**Table 1:** Characteristics of the study population

Characteristics of Women	Categories	n (215)	%
Age	< 30	47	21.9
	30–39	71	33.0
	40–49	40	18.6
	≥ 50	57	26.5
Ethnic group	Arabic	75	34.9
	Kurdish	65	30.2
	Turkish	59	27.4
	Roma	16	7.4
Mother tongue	Arabic	75	34.9
	Turkish	73	34.0
	Kurdish	67	31.2
District/country of birth	Turkey (İzmir)	59	27.4
	Turkey (Other)	73	34.0
	Syria	83	38.6
Marital status	Formally married	137	63.7
	Religious marriage	31	14.4
	Single	50	23.2
Education level	Illiterate	51	23.7
	Literate	26	12.1
	Primary school	57	26.5
	Middle School	40	18.6
	High school and higher	41	19.1
Number of children	0	25	11.6
	1–2	64	29.8
	3–4	75	34.9
	≥ 5	51	23.7
Work status	Homemaker	180	83.7
	Occasional, temporary jobs	22	10.3
	Worker	13	6.1
Work history	Yes	93	43.3
	No	122	56.7
**Characteristics of Husband**		**n (160)**	
Education	Illiterate	19	11.9
	Literate	20	12.5
	Primary school	57	35.6
	Middle School	31	19.4
	High school	30	18.8
	University	3	1.9
Work status	Occasional, temporary jobs	56	35.0
	Unemployed	28	17.5
	Casual employee	27	16.9
	Regular Worker	24	15.0
	Retired	20	12.5
	Own-account worker	5	3.1
Social security	Uninsured	93	58.1
	Insured	67	41.9
**Household**		**n (215)**	
Income	1.-25. percentile (300–900 TL)	55	25.6
	26.-50. percentile (901–1500 TL)	53	24.7
	51.-75. percentile (1501–2500 TL)	70	32.6
	76.-100. percentile (2500–10,000 TL)	37	17.2
Income perception	Poor	117	54.4
	Not good or poor	80	37.2
	Good	18	8.4
Individuals in household	< 3	44	20.5
	3–4	75	34.9
	≥ 5	96	44.7

With a Cronbach’s coefficient of 0.78, the WASH-Behaviors scale was compatible with factor analysis (KMO = 0.805, *p* < 0.05). The scale accounted for 54.3% of the total variance. The calculated Spearman-Brown coefficient for fourteen items was 0.62. Each item’s item-to-total correlation coefficient exceeded 0.30. The WASH-Conditions in Household Scale had a Cronbach’s coefficient of 0.85, and it explained 52.2% of the total variance (KMO = 0.787, *p* < 0.001). The items’ Spearman-Brown coefficient was 0.67, and the item-to-total correlation was greater than 0.3 for each item. As a result, the scale minimum and maximum score were 5–56 and 1–16, respectively. The WASH-Behaviors scale and the WASH-Conditions in Household Scale are displayed in Tables 2 and 3.

**Table 2:** WASH-Behaviors scale items according to factor loadings

Items	Faktorload		
	Factor 1	Factor 2	Factor 3
1. I wash my hands before eating	0,911	-0,085	0,097
2. I wash my hands after eating	0,888	-0,171	0,066
3. I wash my hands before preparing food	0,820	0,004	0,068
4. I wash my hands after going to the latrine	0,670	0,186	-0,180
5. I wash my hands upon returning home	0,588	0,285	0,070
6. Everyone has their own tooth brush at home.	0,094	0,767	-0,088
7. I brush my teeth every day	0,062	0,673	0,174
8. I take a bath at least once a week	-0,108	0,650	0,060
9. Everyone has their own bath towel at home	0,128	0,629	-0,383
10. I change my underwear every day	-0,063	0,561	0,384
11. I flush/pour water after using the latrine	0,211	0,320	-0,002
12. House is swept and cleaned at least once a week	-0,022	0,134	0,740
13. Garbage is thrown out every day	0,140	-0,126	0,739
14. Laundry such as bed linen and duvet covers are washed at least twice a month at 60 °C	0,190	0,198	0,346

**Table 3:** WASH-Conditions in Household Scale items according to factor loadings

Items	Factorload		
	Factor 1	Factor 2	Factor 3
1. Spilling/peeling paint on walls and ceiling	0,775	-0,002	0,041
2. Swelling/depression in the floor	0,704	0,041	-0,054
3. Water leaks on doors/windows	0,628	0,113	0,080
4. Visible mold or mildew odor	0,607	-0,021	-0,076
5. Bathroom inside the house	0,528	-0,010	-0,274
6. Latrine shared with other residences/outside the building	0,485	0,097	-0,349
7. Windowless or darkroom	0,427	-0,212	0,062
8. Evidence of pests or rodents at home	0,373	-0,078	-0,174
9. At least one piped water in the kitchen	0,321	-0,040	0,064
10. Sleeping area per person 3.7 m²	0,028	-0,900	-0,032
11. Population per room over 1.5 people/room	-0,073	-0,871	-0,001
12. 14 m² for one person, 9.3 m² for each other member	0,067	-0,870	-0,046
13. Soap in/near the latrine	-0,014	0,000	-0,929
14. Piped water in/near the latrine	-0,024	0,067	-0,925
15. Toilet paper in the latrine	-0,006	-0,269	-0,590
16. Dustbin/litter bag in home	0,174	-0,269	-0,422

Table 4 shows the relationship between the scores on the two scales and the independent variables. As a result, Roma, women with less than a secondary education, women with more than three children, the unemployed, and women with no work history had lower WASH-Behaviors scores. Average scores were also lower in households with a spouse earning an irregular monthly income, a poor income perception, and more than four family members.

**Table 4:** Score averages of the Scales of WASH according to the sociodemographic characteristics of the household

Characteristics		WASH Behaviors		WASH Conditions	
		Mean + SD	p	Mean + SD	p
Age	< 30	43.70 ± 8.00	0.408^c^	6.81 ± 4.17	**< 0.001** ^d^
	30–39	41.56 ± 7.76		7.23 ± 3.86	
	≥ 40	41.63 ± 9.70		9.54 ± 4.02	
Ethnic group	Turkish	43.89 ± 8.07	**0.001** ^c^	11.03 ± 3.57	**< 0.001** ^d^
	Kurdish	40.40 ± 9.81		8.40 ± 4.11	
	Arabic	43.53 ± 7.73		5.92 ± 3.26	
	Roma	35.18 ± 6.75		7.31 ± 3.94	
Mother tongue	Turkish	41.91 ± 8.49	0.133^c^	10.21 ± 3.98	**< 0.001** ^d^
	Kurdish	40.38 ± 9.84		8.46 ± 4.16	
	Arabic	43.70 ± 7.68		5.95 ± 3.21	
Country of birth	Turkey	42.25 ± 9.00	0.521^a^	9.80 ± 3.97	**< 0.001**
	Syria	41.77 ± 8.35		5.59 ± 3.03	
Marital status	Married	41.74 ± 8.56	0.261^a^	7.86 ± 0.31	**0.031** ^a^
	Single	43.21 ± 9.36		9.32 ± 0.60	
Education level (school)	No	39.31 ± 9.64	**< 0.001** ^b^	7.75 ± 4.10	0.294^b^
	Yes	43.60 ± 7.82		8.41 ± 4.13	
Number of children	≤ 3	44.04 ± 7.65	**< 0.001** ^a^	8.74 ± 4.14	**0.012** ^a^
	> 3	38.72 ± 9.47		7.23 ± 4.07	
Work status	Yes	44.88 ± 5.74	**0.037** ^b^	9.91 ± 4.44	**0.007** ^b^
	No	41.51 ± 9.12		7.84 ± 4.04	
Work history	Yes	43.98 ± 8.16	**0.007** ^b^	9.30 ± 4.40	**< 0.001** ^b^
	No	40.59 ± 8.91		7.32 ± 3.79	
Education level of the husband	< 8 years	39.62 ± 9.44	**< 0.001** ^a^	7.55 ± 4.13	0.469^a^
	≥ 8 years	45.38 ± 5.97		8.10 ± 3.77	
Occupation of the husband	No	40.60 ± 8.68	**0.044** ^a^	6.72 ± 3.78	**0.003** ^a^
	Yes	43.36 ± 8.46		7.57 ± 3.78	
Health insurance	Uninsured	43.74 ± 8.21	**0.017** ^a^	10.54 ± 3.52	**< 0.001** ^b^
	Insured	40.85 ± 8.94		6.47 ± 3.73	
Monthly income	≤ 1500 TL	40.90 ± 9.01	0.069^b^	6.80 ± 3.85	**< 0.001** ^b^
	> 1500 TL	43.23 ± 8.34		9.57 ± 4.03	
Income perception	Poor	39.58 ± 9.48	**0.002** ^c^	6.71 ± 3.74	**< 0.001** ^d^
	Not good or poor	44.19 ± 7.35		8.56 ± 3.98	
	Good	45.13 ± 5.50		10.27 ± 3.71	
Number of households	≤ 4	43.48 ± 8.71	**0.002** ^a^	9.52 ± 3.98	**< 0.001** ^a^
	> 4	40.30 ± 8.50		6.51 ± 3.80	
Parasitic infection	Yes	39.91 ± 9.99	0.354^b^	6.43 ± 4.08	**0.033** ^b^
	No	42.32 ± 8.57		8.39 ± 4.14	
Diarrhea	Yes	39.61 ± 9.42	**0.025** ^a^	7.07 ± 3.66	**0.019** ^b^
	No	42.94 ± 8.34		8.58 ± 4.28	

^a^: Student t test

^b^: Mann Whitney U test

^c^: ANOVA test

^d^: Kruskal Wallis test

WASH-Conditions scores were significantly higher in households with: women aged 40 and up, those who reported their ethnic group as Turkish, those born in Turkey, single, having a job that generates regular income, those with a work history, having spouses who work in a job that generates a regular monthly income, and those with social security. In households with a monthly income of half the minimum wage or less, as well as in households with a low income and more than four household members, the WASH-Conditions score was found to be significantly lower.

**Table 5:** Linear Regression Model Showing Variables Associated with WASH-Behaviors Scores

WASH Behaviors Determination	B	P	Confidence Interval	
			Lower	Upper
N of children	-1.240	< 0.001	-1.941	-0.538
Ethnic group *(Roma)*	7.025	0.002	2.531	11.520
Education level of the husband*(< 8 years)*	3.038	0.022	0.448	5.628
Income perception*(Poor)*	3.327	0.013	0.717	5.938
Diarrhea*(Yes)*	3.007	0.016	0.592	5.562
WASH Conditions	0.723	< 0.001	0.148	0.827
R^2^ = 0.48 *p* < 0.001				

According to multiple linear regression analysis, the WASH-Behavior score decreased by a factor of 1.2 for each additional child in the household. Arabs, Kurds, and Turks are more likely to engage in WASH-Behaviors than Roma. Women were more likely to be unable to meet their WASH needs if their perceived income is low, if there was a case of diarrhea in the household, and if the education level of their spouse is below secondary school. Independent variables explained 48% of the change in WASH-Behaviors scores (Table 5).

## Discussion

The present study aimed to follow an approach that evaluates WASH holistically in a socioeconomically disadvantaged community. Although it was carried out in a very poor area, the study revealed that there was no remarkable inadequacy as regards WASH-Behaviors; nevertheless, the current conditions were inadequate in terms of providing all three components of the structure of WASH. This study conducted in a city center revealed results that exposed the problems experienced by the poorest and most disadvantaged groups in terms of the triad of WASH, one of the main determinants of health.

Two separate scales were developed to work within the framework that analyzed the triad of water, sanitation, and hygiene in relation to one another as well as in terms of household member behaviors and current house conditions. These scales were found to be successful in terms of validity and reliability. However, the fact that the household conditions score was lower than the behaviors in determining the WASH level suggests that it is a more realistic indicator in determining poverty [16]. Similarly, the study discovered that WASH cannot be defined solely in terms of behaviors or conditions; these two components must be considered together, even if the assessment methods and determinants of behaviors and conditions differ.

Due to the sociocultural status of our study area, our findings only apply to women. However, studies in the literature suggest that women carry a disproportionate share of the burden for WASH in groups of comparable socioeconomic status [17, 18]. Furthermore, our findings revealed that the research group exhibited characteristics such as low income, a history of immigration, overcrowded housing, unemployment, insecurity, and a lack of education. The average monthly income per household was below the hunger threshold when the minimum wage and average number of family members in each household were considered [19, 20]. The women and their husbands had a lower level of education when compared to the adult population of Turkey [20]. Women’s employment rate was roughly half those of the Turkish average, and household heads’ unemployment rate was double those of the Turkish statistics [19, 21]. The average number of people living in households (4.5) was also higher than Turkey’s average household size [19]. As a result, the people of the Basmane district, where disadvantages such as poverty, migration, overcrowding, lack of education, unemployment, and gender roles coexist, reflect the vulnerable and poor communities of cities.

In comparison to Turkish data, the study population’s hand hygiene was mostly good, but body and household hygiene were lacking [22]. Handwashing practices in the research group were better than would be expected for a group with similar disadvantages. Nonetheless, this finding could be attributed to the fact that all of the participants in the study were female. According to studies, women wash their hands more frequently after using the restroom than men [23, 24]. Another reason for the high frequency of hand washing may also be attributed the fact that women might have exaggerated when they reported their hygiene behaviors. Because data collection occurred during the peak of the COVID-19 pandemic, increased illness anxiety may have resulted in higher rates of hand washing, in addition to reporting bias [9, 25].

Although multivariate analysis does not support it, bivariate analysis discovered ethnicity to be a significant predictor of WASH level. Those who identified as Roma had the poorest WASH-Behaviors, whereas those of Turkish origin had better WASH-Conditions than other groups. Roma communities in Europe have been reported to face more barriers to WASH than majority populations, affecting their hygiene behaviors [26]. Our multivariate analyses yielded comparable results. As a result, the Roma, one of Turkey’s most vulnerable ethnic minority groups, appear to be affected by poverty and unemployment in terms of WASH-Behaviors.

While total monthly income was unrelated to WASH behaviors, there was a strong relationship with income perception. This could be because the absolute income level of all households in the research group is very low and similar, the interviewed women do not know their exact income level, some of them receive financial assistance, albeit not on a regular basis, and perception reflecting the relative situation better reveals differences within the same community. Similarly, as the education level of women and their husbands increased, so did their score of the WASH Behavior scale. However, hygiene behavior increases with income perception regardless of education level. Higher socioeconomic status was also found to have a higher level of personal and domestic hygiene in India [27]. Poor and vulnerable populations had less access to improved WASH services, and their associated behaviors were worse [17, 26].

It has been determined that living in overcrowded households worsens both WASH-Behaviors and conditions. The fact that most households consisted of nuclear families suggests that the number of children may be the most influential factor in determining the number of people living in a household. Multivariate analyses also supported this finding. Domestic responsibilities impose time constraints on women, preventing them from engaging in adequate hygiene behaviors [27]. A study conducted in Zambia found that households with fewer than four people had a higher level of WASH, which is consistent with our findings [28].

There was a strong link between household socioeconomic characteristics and WASH-Conditions. WASH-Conditions were significantly influenced by monthly income level and perception of income. The contribution of women to a household’s total income can explain the high level of WASH-Conditions among working women as well as those who worked in the past. The fact that the husbands of these women also worked in jobs that provided regular income and were covered by social security had a positive effect.

Despite the poor socioeconomic characteristics of the study group, WASH-Behavior was found to be relatively good, but conditions were problematic. Studies have proven the impact of sanitation factors, such as toilet, tap and soap, on the hygiene behavior [29, 30]. These variables were utilized as socioeconomic indicators, specifically indicating access to water [16, 31]. Because of this, one of the fundamental WASH interventions should be ensuring that households have access to running water and soap. It would be beneficial to evaluate the conditions and behaviors together while determining the minimum level of WASH interventions to be undertaken in disadvantaged groups.

Although the use of a newly developed scale could be seen as a limitation, our validity and reliability analyses produced successful results. It should be noted that the research area was limited in terms of representing urban poverty in regions where migration and population dynamics are lower, and the findings include the possibility of reporting bias. Furthermore, its validity should be evaluated before being used in populations where men share responsibility for WASH in households.

## Conclusion

This study’s scale development process emphasized the importance of taking into account both behaviors and household conditions, albeit using separate techniques. The resulting behavior and household conditions scales were found to be valid, and their scores were related to basic sociodemographic and economic characteristics such as education, income, household size, and ethnicity. In contrast, the study discovered that conditions, rather than behaviors, are problematic in WASH, and that behavioral interventions will not work unless the conditions are corrected. With this result in mind, the municipality was advised to replace mains water pipes in the Basmane area, collect garbage more frequently, and implement other housing-improvement measures. Furthermore, the fact that women bear the majority of the burden of home care in the research area highlights the importance of addressing the WASH issue from a gender perspective in studies conducted in regions with similar socioeconomic structures.

## Data Availability

The data that support the findings of this study are available upon request from the corresponding author.
